# Diffusion and dissemination of evidence-based dietary strategies for the prevention of cancer

**DOI:** 10.3390/curroncol13040014

**Published:** 2006-08

**Authors:** D. Ciliska, P. Robinson, T. Horsley, P. Ellis, M. Brouwers, M. Gauld, F. Baldassarre, P. Raina

**Affiliations:** * School of Nursing, McMaster University, Hamilton, Ontario; † Department of Clinical Epidemiology and Biostatistics (CEB), McMaster University, Hamilton, Ontario.; ‡ Cancer Care Ontario Program in Evidence-based Care, McMaster University, Hamilton, Ontario.; § Hamilton Regional Cancer Centre, Hamilton, Ontario.; || McMaster University Evidence-based Practice Center, Hamilton, Ontario

**Keywords:** Information dissemination, dietary habits, review, cancer control interventions

## Abstract

We used a systematic review to identify strategies that have been evaluated for disseminating cancer control interventions that promote the uptake of a healthy diet in adults. Studies were identified by contacting technical experts and by searching medline, PreMedline, cancerlit, embase/Excerpta Medica, Psycinfo, cinahl, the Cochrane Database of Systematic Reviews, and reference lists. English-language primary studies were selected if they evaluated the dissemination of healthy diet interventions to individuals, health care providers, or institutions. Studies involving only children or adolescents were excluded.

We retrieved 101 articles for full-text screening, and identified nine reports of seven distinct studies. Four of the studies were randomized trials, one was a cohort design, and three were descriptive studies. Six of the studies were rated methodologically weak, and one was rated moderate. Because of heterogeneity, low methodological quality, and incomplete data reporting, the studies were not pooled for meta-analysis. No beneficial dissemination strategies were found. One strategy involving the use of peer educators at the work site, which led to a short-term increase in fruit and vegetable intake, looks promising.

Overall, the quality of the evidence is not strong, and the evidence that exists is more descriptive than evaluative. No clear conclusions can be drawn from these data. Controlled studies are needed to evaluate dissemination strategies and to compare dissemination and diffusion strategies that communicate different messages and target different audiences.

## 1. INTRODUCTION

It has been estimated that one third of all cancer mortality in the United States is related to diet^1^. Reviews of dietary studies have led groups such as the American Institute for Cancer Research to recommend that diet should largely be based on plant products, with 400 g in vegetables and fruits providing more than 10% of the energy consumed daily^2^,^3^. The American Cancer Society added that intake of high-fat foods and alcohol should be limited^4^.

National objectives in both the United States and Canada have been set at 5 or more servings per day of fruits and vegetables^5^. Average intake falls considerably short of this objective. In the United States, intake is estimated to be 3.4 total servings of fruits and vegetables per day on average, but that estimate differs by age, ethnicity, and socioeconomic status^6^.

Considerable recent research has focused on dietary change to increase fruit and vegetable consumption and to reduce fat consumption. The effectiveness of these interventions has been the subject of several systematic reviews^7^.

Some evidence suggests that educating physicians in dietary counselling is an effective dietary intervention. However, there is no consistent evidence of the effectiveness of other interventions directed at health care providers. Interventions directed at individuals that were shown to have some effect in producing dietary change include tailored interventions, multiple interventions and multiple contacts, and environmental interventions. Media campaigns may result in increased knowledge and awareness of behaviours that reduce risk^7^.

As the evidence grows for the effectiveness of dietary interventions, more attention is expected to be given to the dissemination and diffusion of these interventions so as to promote dietary change. The theoretical background for research dissemination and diffusion is complex and often contradictory. Theoretical bases and models for dissemination and diffusion of research in general and for behaviour change among health care practitioners and the public are available. These major fields of dissemination and diffusion, and practitioner and client behaviour change, are inconsistently integrated into the development of interventions, and the field of cancer control is no exception.

Closing the gap from knowledge generation to use of knowledge in decision-making for practice or policy is conceptually and theoretically hampered by diverse terms and inconsistent definitions of terms, including diffusion, dissemination, knowledge transfer (or translation or uptake or utilization), adoption, and implementation. Research rarely distinguishes between interventions to change behaviour and strategies to disseminate the interventions. Furthermore, many studies have combined their evaluation of interventions and strategies. Some activities (for example, media campaigns, opinion leaders, and peer educators) can be characterized as both cancer control interventions and strategies to disseminate cancer control interventions to target audiences. This characterization can lead to confusion about what is considered a cancer control intervention and what is considered dissemination of cancer control interventions.

For the purpose of this evidence report, if an activity was used to provide information about the benefits of a desired cancer control behaviour, it was classified as a cancer control intervention. If the activity was used to provide information about the availability or benefits of a cancer control intervention, it was classified as a strategy to disseminate a cancer control intervention.

In keeping with the views of Lomas^8^, this evidence report uses the term “dissemination” to refer to the active process of transferring cancer control interventions to target audiences and “diffusion” to refer to the passive spread of cancer control interventions.

## 2. MATERIALS AND METHODS

This review addresses the question “What strategies have been evaluated to disseminate cancer control interventions that promote the uptake of a healthy diet in adults?”

We systematically reviewed primary studies of dissemination and diffusion strategies for dietary interventions. We did not include studies of the effectiveness of direct interventions to change dietary intake; rather, we included studies focused on the dissemination of interventions to adults and health care professionals.

Primary studies were considered for inclusion if they were published in English during ?1980 or later, and if they evaluated dissemination of a cancer control intervention in one of five topic areas. All primary studies, regardless of study design, were eligible for inclusion. Reports focused exclusively on children or adolescents were excluded.

Search strategies were developed as an iterative process in consultation with the librarian at the McMaster Evidence-based Practice Center. The search strategy can be consulted online at www.ahrq.gov/clinic/tp/cancontp.htm (report name: *Diffusion and Dissemination of Evidence-based Cancer Control Interventions*; file name: 27appc.doc).

Similar databases were also searched for both objectives:

- medline, the U.S. National Library of Medicine database
- PreMedline
- cancerlit
- embase, the Excerpta Medica database
- Psychinfo
- The Cumulative Index to Nursing and Allied Health Literature (cinahl)
- Sociological Abstracts
- HealthStar
- Cochrane Database of Systematic Reviews

Reference lists of pertinent articles and reviews were also searched, and technical experts were consulted.

All data extraction forms were developed, pilot-tested, and revised by members of the local research team. Two reviewers completed data extraction independently for all reports. Disagreements that arose were resolved by consensus. The research team discussed differences that could not be resolved by the reviewers. Quality assessment was undertaken using standardized quality assessment tools developed by the Effective Public Health Practice Project. Tables were constructed to describe the most salient characteristics of the eligible studies. Meta-analysis was not undertaken because the studies varied substantially in terms of study design, interventions assessed, outcome measurements, methodologic quality, and completeness of data reporting.

The present report therefore represents a systematic narrative review of the existing evidence.

## 3. RESULTS

### 3.1 Included Studies

**FIGURE 1:**
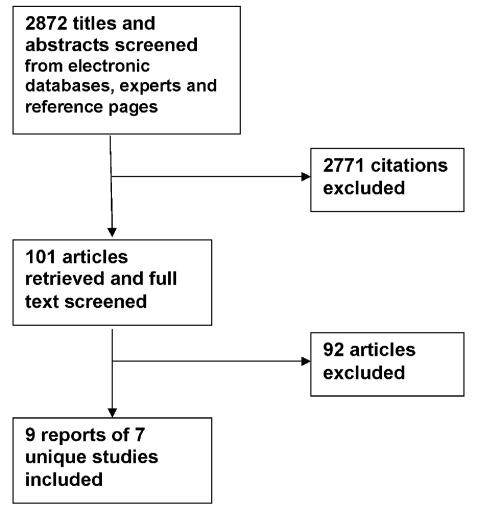
Adult health diet: search yield for studies evaluating dissemination strategies.

The electronic database search identified 2872 articles; 101 were retrieved for full text screening (Figure 1). Of these, 9 reports of seven distinct studies are included: 3 reports about one study^9^–^11^, and reports of six other studies^12^–^17^. The latter six studies are presented in Table I. We excluded 92 articles for lack of relevance; they did not address dissemination and diffusion strategies for dietary interventions.

Although the search inclusion criteria were broad, all of the eligible studies were conducted in the United States. Six reports had been published after 1998; the other four had been published between 1989 and 1993. All seven projects were funded: five^9^,^14^–^17^ by the National Cancer Institute (nci), one 12 by the National Institutes of Health, and one^13^ by a private foundation.

One study achieved a rating of “moderate”^14^; all others were “weak” as defined by the standardized assessment tool^18^. The tool was adapted from those developed by Clarke *et al.*^19^ and Jadad *et al.* 20

Because community interventions are often not evaluated by randomized trials, the tool reflects other possible study designs, and rates these criteria:

- Selection bias
- Study design
- Confounders
- Blinding
- Data collection methods (reliability and validity)
- Withdrawals and dropouts
- Intervention integrity
- Analyses

Based on a dictionary and a standardized guide to assessing component ratings, each component was rated “strong,” “moderate,” or “weak.” Content and construct validity had been established (Thomas H, *et al.* 2001, unpubl. data). A comparison of the tool used in this review was made with the tool used in the *Guide to Community Preventive Services* 21.

Four of the studies were randomized trials 9,14,16,17; none of the other studies included a comparison group. Three articles were descriptive 11,13,15, and one article was a cohort study 12 (Table I). The included studies were diverse in regard to the intervention disseminated and the strategies used for dissemination and diffusion. Only two studies compared two strategies 16,17. Of these, one study 17 compared a training workshop to postal delivery of information. The second study 16 evaluated whether the use of educational facilitators (academic detailing) plus a workshop was more effective than educational facilitators (academic detailing) only. Each of the other studies evaluated the effectiveness of a single dissemination strategy. One strategy assessed was “train the trainer” to disseminate preventive-medicine education to physicians 12; two studies evaluated media campaigns for promoting access to a telephone information service 13,15; one study assessed the effect of peer educators for improving fruit and vegetable consumption 9–11; and one looked at the dissemination of intervention materials to control sites following completion of a work site nutrition intervention 14.

Outcomes were diverse across studies and were not usually behavioural outcomes but rather process indicators, such as the number of training sessions conducted 12, the number of physicians trained^12^, the number of consumer telephone calls^13^,^15^, a count of peer-education strategies according to sex and ethnicity^11^, and uptake of materials by control sites after an intervention^14^. Client-based outcomes included knowledge^12^ and intake of fruits and vegetables^9^,^10^.

### 3.2 Dissemination Studies That Targeted Health Care Providers

#### 3.2.1 Train the Trainer

One “train-the-trainer” study aimed at disseminating preventive-medicine education to physicians^12^. Faculty from general internal divisions across the United States were invited to apply for a month-long Stanford Faculty Development Program; 10 were chosen and trained to be Clinical Preventive Medicine facilitators. They then went to their home institutions and trained other faculty at their home site.

Fidelity checks concluded that the facilitators adhered closely to the curriculum that they had been taught. The medical faculty educated by the facilitators had an increase in knowledge and self-efficacy to use behaviour changes to promote a healthy diet. Subsequently, house staff physicians interacting with faculty who had attended the facilitator-run sessions reported an increase in the degree of preventive-medicine content in teaching interactions and an increase in their ratings of self-efficacy to implement preventive-medicine strategies.

Although the train-the-trainer model shows some promise, it needs to be evaluated with a more rigorous design. Furthermore, many biases are likely to be inherent in the selection of internists who were able to leave their work situation for a month of training.

#### 3.2.2 Academic Detailing (Educational Facilitators)

One randomized control trial (rct)^16^ used academic detailing to target dissemination to health care providers. In this trial by Dietrich *et al.*, primary-care medical practices were randomized to one of four groups: facilitator only, facilitator plus workshop, workshop only, or a control group. Practices in the facilitator-only group (*n* = 24) received 3–4 visits from a facilitator who provided detailed instruction and assistance in selecting and implementing non-computer-based office-system interventions. Practices in the facilitator-plus-workshop group (*n* = 26), not only received visits from an educational facilitator, but also sent a physician from the practice to attend a one-day workshop. The workshop session reviewed the nci’s prevention and screening recommendations, but did not provide information on the use of office-system interventions. Practices in the workshop-only group (*n* = 24) attended the workshop. Practices in the control group (*n* = 24) received no information.

Cross-sectional patient surveys were conducted before randomization and again at the 12-month follow-up. The study reported on two diet-related outcomes:

- The number of patients reporting that their physician had advised them to reduce their fat intake
- The number of patients reporting their physician had advised them to increase their fibre consumption

At the 12-month follow-up, significantly more eligible patients in the facilitator-only group than in the control group reported that their physician had advised them to reduce their fat intake (0.56 vs. 0.47, *p* < 0.05). There was no significant difference at 12-month follow-up between the facilitator-plus-workshop group and the control group in the number of patients reporting advice to decrease fat intake (0.51 vs. 0.47). At the 12-month follow-up, there was no significant increase in the number of eligible patients in the facilitator-only or facilitator-plus-workshop groups as compared with the control group reporting advice to increase fibre consumption (facilitator vs. control: 0.48 vs. 0.38; facilitator-plus-workshop vs. control: 0.41 vs. 0.38). The overall conclusion from this trial was that the use of educational facilitators to disseminate and implement office-system interventions could improve the provision of prevention and early detection services in community practices.

The use of educational facilitators (academic detailers) to disseminate office-system interventions appears to be a promising strategy. Further research in this area is needed.

#### 3.2.3 Workshops

The rct by Tziraki *et al.* 17 assessed the effectiveness of two strategies for promoting the use by primary care physicians and their office staff of an nci nutrition manual. The nutrition manual was modelled after the nci publication *How to Help Your Patients Stop Smoking.* Medical practices randomized to the workshop group (*n* = 244) were invited to send one staff member to a 3-hour training workshop on how to use the nutrition manual. Training was provided in four major components of the manual:

- How to organize the office environment
- How to screen for patient adherence
- How to provide dietary advice
- How to implement a patient follow-up system

Medical practices assigned to the postal-delivery group (*n* = 256) received the nutrition manual in the mail with no further information. Medical practices in the control group (*n* = 255) did not receive the nutrition manual.

Follow-up interviews with medical staff and observational assessments were conducted at 4–6 months after dissemination of the manual. Adherence scores were calculated for four areas: office organization, nutrition screening, nutrition advice or referral, and patient follow-up. The workshop session drew low attendance; fewer than 50% of the assigned practices sent representatives (120 of 244). The authors of the trial used an “intent to treat” approach for the primary statistical analysis and included all practices in the workshop group regardless of attendance.

The workshop group was significantly more adherent to the manual’s recommendations for office organization at follow-up than either the postal-delivery group (28.5% vs. 24.7%, *p* < 0.005) or the control group (28.5% vs. 23.0%, *p* < 0.001). Of those practices who sent a representative to the workshop, 30.6% were adherent to the recommendations for office organization. No significant difference was observed between the postal-delivery group and the control group for office organization (24.7% vs. 23.0%).

The workshop group was also significantly more adherent to the manual’s recommendation for nutrition screening than either the postal-delivery group (23.5% vs. 21%, *p* < 0.05) or the control group (23.5% vs. 20.5%, *p* < 0.05). Of practices that sent a representative to the workshop, 25% were adherent to the nutrition screening recommendations. No significant difference was observed between the postal-delivery group and the control group for nutrition screening (21% vs. 20.5%).

There was no statistically significant difference between the three groups for providing nutrition advice (workshop: 54.9%; postal delivery: 53%; control: 52.3%), nor for patient follow-up (workshop: 14.6%; postal delivery: 13.6%; control: 13.6%). A secondary analysis showed that practices that had sent a representative to the workshop were significantly more likely than either the postal-delivery group (57% vs. 53%, *p* < 0.05) or the control group (57% vs. 52.3%, *p* < 0.05) to provide nutrition screening. No significant difference was observed for patient follow-up on secondary analysis.

Training workshops appear to hold some promise as a dissemination strategy; however, motivating medical professionals to attend these sessions may be a difficult barrier to overcome. Further research in this area is needed.

#### 3.2.4 Postal Delivery

One rct 17 evaluated the effectiveness of postal delivery as a dissemination strategy. This trial compared the effectiveness of postal delivery with a training workshop to disseminate an nci nutrition manual to primary care practices. Postal delivery was not found to be an effective method to disseminate the nutrition manual. Refer to 3.2.3, “Workshops” for the detailed results of the study.

### 3.3 Dissemination Studies That Targeted Work Sites

#### 3.3.1 Passive Dissemination

The Working Well Trial 14,22 randomized 114 work sites representing more than 28,000 workers to test the effectiveness of health promotion activities that were planned and delivered with a high level of employee participation. The intervention phase lasted for 2 years, and then nutrition materials were disseminated to the control sites, followed by a further 2-year assessment. The investigators were particularly interested to see if the control sites would use the materials. No information was given about the actual strategies used to get the nutrition intervention materials to the control group, nor was any measure of uptake reported. No changes occurred in the level of nutrition activities in the control sites.

#### 3.3.2 Peer Educators

An opinion-leader strategy was tested using peer educators in the work-site intervention called “5-A-Day: Healthier Eating for the Overlooked Worker.” Although rated methodologically weak, this rct holds promise as an area for further research. The 5-A-Day intervention aimed to increase fruit and vegetable consumption in an ethnically mixed population of 2091 lower socioeconomic labour and trade employees 9,10. The intervention group and the control work sites both received an 18-month intervention program of educational materials through workplace mail, cafeteria promotions, and speakers. In the intervention group, naturally occurring “cliques” were identified, and within those cliques, ratings were given to each individual regarding their degree of “centrality” to communication ties and flow. People rated highest in “centrality” became the peer educators for their clique, mimicking the “opinion-leader” strategy.

Peer educators attended a 16-hour training program, where they were given information about the health benefits of eating fruits and vegetables, cultural trends in dietary practices, a peer educator’s roles and responsibilities, five persuasive communication strategies (foot-in-the-door, fear appeal, benefits, peer pressure, and questioning), and ways to initiate informal conversations about fruits and vegetables. They were instructed to engage in nutrition education of their co-workers for about 2 hours per week, on work time. They also distributed 5-A-Day materials produced specifically for this population: a nine-booklet resource guide, four issues of a newsletter, enabling gifts such as a recipe book, and vegetable seeds. The peer educator intervention lasted 9 months, with consumption measured at the end of the intervention and at a 6-month follow-up.

The result was an increase in fruit and vegetable consumption of 0.77 total servings per day more in the intervention group than in the control group (measured by recall, *p* < 0.001) and an increase of 0.46 total daily servings (measured by food frequency, *p* < 0.002) 9. The effect was maintained at the 6-month follow-up for intake recall (increase of 0.41 daily servings, *p* = 0.034), but not for food frequency 9.

In an analysis of the frequency and duration of peer-education contact with co-workers, greater contact with the peer educators was related to larger immediate increases in fruit and vegetable intake, particularly vegetable intake, but was not related to total intake at the 6-month follow-up 10. A qualitative design, used to study the educational strategies used by the peer educators in the intervention group, found that these strategies varied by sex and ethnicity 11. Hispanic educators were more likely than non-Hispanic educators to use individual rather than group change strategies; and men more frequently used strategies such as “mock competition,” and “giving materials” and “encouragement”; female peer educators more often used “creating context,” and “keeping 5-A-Day visible” 11.

Few work site dissemination strategies have been evaluated. In one, the dissemination strategy was not evaluated 14. The other study, using an opinion-leader strategy, had at least a short-term impact on consumption.

### 3.4 Dissemination Studies That Targeted Individuals

#### 3.4.1 Media Strategies

Two studies evaluated multiple media channels (print, television, radio) to assess the impact of media campaigns on telephone calls to an information telephone line 13,15. Project lean (Low-Fat Eating for America Now) was a 3-year initiative, begun in 1989, to reduce dietary fat consumption. The media campaign led to hotline access of 300,000 consumer calls in 18 months (25,000–28,000 calls per month), but the calls declined as publicity declined, and the line was terminated because of expense, estimated to be US$300,000 annually 13.

Although these outcomes were not assessed in a direct comparison, some important lessons were learned in this study. Well-placed advertising may be the most appropriate and effective communications strategy for a national nutrition social marketing campaign because it can more easily be tailored to the particular audience than public service announcements can. It can also communicate information more directly and reduce the need for an information hotline or follow-up materials. Furthermore, building a network of state and local programs and partnerships with the food service industry can allow a campaign to reach a broader audience 13.

A second primary study was identified in which calls to the Cancer Information Service (cis) hotline were analyzed. Callers were asked “How did you first find out about the cis?” Records of a subsample of people (214,472) who inquired about smoking, nutrition, Pap smears, and breast self-evaluation were reviewed. Television was the most frequently reported source of learning about the information line, regardless of age, sex, or ethnic group. An exception was the group of callers of Asian or Pacific heritage; they reported publications as the more common source of information about the hotline 15.

Media dissemination strategies, particularly those using television, can make people aware of information lines and prompt them to call. However, from these two studies, it appears that hotlines are expensive to advertise and maintain.

## 4. DISCUSSION

Recognition of the need for processes to transfer new knowledge into routine practice is increasing. Traditional methods of knowledge transfer such as journals and conferences have not proven effective in changing behaviour 23. Emphasis has been placed on the importance of research examining the dissemination of evidence-based knowledge and its uptake by the targeted recipients. Target audiences include providers, policymakers, and the general public.

The present review has several limitations. It does not address the effectiveness of the dietary interventions themselves—just the dissemination interventions used to lead others to learn about the dietary interventions. The results and conclusions are based on information available in published English-language reports. Contact with the authors may have compensated for any reporting difficulties that resulted in a lower quality rating for the studies. Meta-analysis was deemed inappropriate because of the diversity in the target groups, interventions, and outcome measures.

Few studies of the dissemination of dietary interventions for cancer prevention have been conducted. Overall, the quality of the evidence is not strong, and it is primarily descriptive rather than evaluative. Either process measures (number of calls, number of physicians educated, or number of education sessions held) are reported or outcomes result from non-validated self-report measures. Controlled studies need to be conducted for dissemination strategies, and dissemination and diffusion strategies with different messages and different target audiences need to be compared. More studies of strategies such as those using opinion leaders or academic detailing should be conducted with health care providers. The idea of a peer educator who is identified more as an opinion leader warrants further exploration. Cost-effectiveness needs to be established for any interventions.

Most of the research on healthy diet and cancer has focussed on evaluating interventions to promote behaviour change. Information on how to disseminate these findings to the community is lacking. Questions to address in future research include these:

- What is the effectiveness of strategies that remind health professionals to give interventions during patient encounters?
- What innovative technologies can be brought to dissemination strategies?
- Once media strategies have alerted the public to services, can effective interventions then be disseminated to individuals in such a way that they will use them to change dietary habits?
- Is there an effective combination or sequencing of strategies that will result in dietary change?
- What policy-level strategies are effective in promoting dissemination of healthy diet interventions?
- What maintenance strategies can be incorporated to maintain the uptake and use of the evidence?

## Figures and Tables

**TABLE I:** Strategy for dissemination of cancer control interventions in adult healthy diet

*Reference*^a^
Albright *et al.*, 1992 12
*Purpose:* Test a dissemination model for providing clinical preventive medicine (cpm) training to internal medicine faculty across the country
*Study design:* Cohort, one group, pre/post (*n=*10)
Internists were trained as trainers; 91 faculty members attended home-site seminars led by trainers
*Target group:* General internal medicine faculty
*Quality assessment:* Weak
*Strategy evaluated:* Train-the-trainer (month-long training, off site)
*Interventions:*cpm curriculum encompassed: smoking cessation, clinical nutrition, weight control exercise, hyperlipidemia, and national screening guidelines
Curriculum provided content on clinical teaching and medical decision-making
Clinical nutrition section included interventions that promoted healthy diets in adults
*Findings:* Fidelity ratings obtained from videotapes of the home-site seminars given by the trainers indicated that the trainers adhered closely to the cpm
Trainers teaching home-site internal medicine faculty: knowledge of smoking cessation, cholesterol, and screening-specific information of faculty members significantly increased post-seminar in the last 2 of the 3 years studied (*p<*0.02 and *p<*0.001)
Faculty’s ratings of their self-efficacy to implement the cpm practices for clinical nutrition demonstrated significant post-test increases for all 3 years (*p<*0.0001)
Faculty use of specific behaviour-change interventions (diaries, self-help materials, and social support) to promote healthy diet increased (*p<*0.05)
85% of the faculty in 1986, 96% in 1987, and 84% in 1988 reported teaching the cpm strategies to their house staff
House staff reported significant increases in the degree to which the faculty addressed cpm in their teaching interactions (1987: *p<*0.0001; 1988: *p<*0.04)
House staff ratings of their self-efficacy to implement specific preventive medicine strategies increased in both years (1987: *p<*0.0001; 1988: *p<*0.002)
Anderson *et al.*, 1989 15
*Purpose:* Examine inquiries received by the cis, a telephone hotline, to determine:
(1) effects of different media in stimulating calls to the cis
(2) demographic characteristics of callers in four cancer prevention and early detection subjects: smoking, nutrition, Pap smear screening, and breast self-examination
*Study design:* Descriptive study
Retrospective analysis of 5 years of inquiries to 1 national and 26 local cis offices in four subject areas; a standardized call record form was completed for each call
Demographic information was collected only during the last 2 years of the study for first-time, non-health professional callers and was limited by federal stipulations to 20% of callers in five cis offices (*n=*57,374) nutrition-related calls over the 5 years studied
*Target group:* Smokers in the United States, Mexico, and other countries
*Quality assessment:* Weak
*Strategy evaluated:* Multiple media sources (television, radio, newspapers)
*Interventions:*cis, a telephone-based information and education program of the National Cancer Institute
*Findings:* Sources of callers’ learning about the cis hotline were television 33.9%, radio 28.2%, publications (including newspapers, magazines, pamphlets, and posters) 26.9%, health care providers 4.3%, significant others 4.6%, and telephone assistance 2.2%
Television was the most common information source reported by callers for both sexes (72.2% of male callers and 60.7% of female callers)
An inverse relationship was found between frequency of television cited as an information source and the age and education of callers. In the 19-year-old or younger age group 81.7% of callers cited television, as compared with 39.6% of callers in the 60-year-old or older age group
Television was the predominant source for four of the five ethnic groups (Caucasians, African Americans, Hispanics, and Native Americans). For callers of Asian or Pacific Island heritage, the most frequently cited source was publications (46.7%), followed by television (32.1%).
Buller *et al.,* 1999 9
*Purpose:* Test a peer-education strategy to promote the 5-A-Day message
*Related papers:* Buller *et al.,* 2000 10 (reported below) and Larkey *et al.*, 1999 11 (reported below)
*Study design:* Randomized controlled trial
Experimental group (*n=*505) employees (in 46 cliques) assigned to receive 5-A-Day peer-education, plus the general 5-A-Day program
Control group (*n=*492) employees (in 46 cliques) assigned to receive the general 5-A-Day program (cafeteria promotions and workplace mail) only
*Target group:* Anglo and Hispanic worksite population in the United States (lower socioeconomic labour and trade employees)
*Quality assessment:* Weak
*Strategy evaluated:* Peer educators (*n=*42)—chosen for “centrality,” rated highest by peers in communication ties and flow—attended a 16-hour training program; trained in persuasive communication techniques
Expectation was that they would spend 2 hours weekly discussing fruit and vegetable intake with coworkers
*Interventions:* 5-A-Day message plus accompanying 5-A-Day printed materials
*Findings:* Immediate changes post-intervention in awareness, attitudes, and dietary behaviour:
Employees receiving peer education increased their awareness of the 5-A-Day program (*p<*0.001) and their knowledge of the 5-A-Day concept, attitudes toward fruit and vegetable intake (*p=*0.024–<0.001)
Number of daily servings of fruits and vegetables consumed increased (0.77 on 24-hour intake recall, *p<*0.001, and 0.46 on food frequency questionnaire items, *p=*0.002)
Persistence of changes in awareness, attitudes, and dietary behaviour (6-month follow-up):
General persistence of the statistically significant increases in the peer-education group, but of reduced magnitude for knowledge of the 5-A-Day program and diet-related attitudes
Statistically significant increases total number of daily servings persisted when measured by 24-hour intake recall (0.41, *p=*0.034), but not by food frequency questionnaire
Buller *et al.,* 2000 10
*Related papers:* Buller *et al*., 1999 9 (reported above) and Larkey *et al.*, 1999 11 (reported below)
*Study design, Target group, Quality assessment, Strategy evaluated, Interventions:* Same study as above
*Findings:* Findings pertain only to the peer-education (experimental group):
57% of employees reported printed materials stimulated discussion of fruits and vegetables with co-workers during program, 31% still discussing 6 months later, 69% discussed printed material with a family member during intervention
Greater contact with peer educators was related to larger immediate increases in total consumption of fruits and vegetables (*p=*0.003) as measured by food frequency items
When food types were examined separately, peer-educator contact was positively related to immediate increased vegetable intake (*p=*0.002), but not to fruit or juice intake
The more employees reported reading the printed material, the smaller the observed immediate increase in fruit consumption (*p=*0.002)
There was no significant association between peer contact and changes in total intake of these foods at the 6-month follow-up
Dietrich *et al.,* 1992 16
*Purpose:* Test the impact of physician education and facilitator assisted office-system interventions on cancer early detection and preventive services
*Study design:* Randomized controlled trial
98 of the 102 practices that agreed to participate completed the study
Unit of randomization was the practice as represented by one physician
Four groups:
Facilitator only (*n=*24 practices)
Workshop-plus-facilitator (*n=*26 practices)
Workshop only (*n=*24 practices)
Control (*n=*24 practices)
*Target group:* Office-based general practitioners and general internists in New Hampshire and Vermont
*Quality assessment:* Weak
*Strategy evaluated:*
(1) Facilitators visited each practice 3–4 times over 3 months for approximately 120 minutes each time Performed an initial audit of each practice to assess the status of preventive care and assisted practices in the design and implementation of office system interventions; practices only implemented those interventions that meet their perceived needs
(2) Facilitator-plus-workshop was the same as (1) plus physician from each practice attended a 1-day workshop led by an expert who reviewed nci’s prevention and screening recommendations and taught specific skills; also provided a written syllabus
Note: The workshop-only and the control groups did not receive information on the use of office-systems interventions for cancer prevention or early detection
*Interventions:* Multiple office-system interventions including preventive-care flow sheets, chart stickers, health education posters and brochures, and patient health diaries (none of the interventions were computer-based)
*Findings:* Response rate for the cross-sectional survey was 91% (*n=*2436 patients) pre-experiment and 93% (*n=*2595) at 12-month follow-up
More eligible patients in the facilitator-only group reported their physician had advised them to reduce fat intake compared to patients in the control group at 12-month follow-up (proportion: 0.56 vs. 0.47, *p<*0.05; baseline results were used as covariates)
No significant increase in the number of eligible patients in the facilitator-plus-workshop group reporting their physician had advised them to reduce fat intake compared with patients in the control group at 12-month follow-up (proportion: 0.51 vs. 0.47)
No significant increase in the number of eligible patients in the facilitator-only or facilitator-plus-workshop groups reporting their physician had advised them to increase fibre consumption compared to patients in the control group at 12-month follow-up (proportion: 0.48 facilitator-only vs. 0.38 control; 0.41 facilitator-plus-workshop vs. 0.38 control)
*Overall conclusion:* Community practices assisted by a facilitator in the development and implementation of an office system can substantially improve provision of cancer early detection and preventive services
Larkey *et al.,* 1999 11
*Purpose:* Assess which persuasive strategies are used by peer health educators, which strategies are used more in one-on-one vs. group contexts, and which strategies are most likely to be used by males and by females
*Related papers:* Buller *et al.,* 2000 10 (same project, see additional findings above) and Buller *et al.,* 1999 9 (same project, see additional findings above)
*Study design:* Qualitative, descriptive study of peer-educator within original randomized trial
*Target group:* Anglo and Hispanic worksite population in the United States
*Quality assessment:* Weak
*Strategy evaluated:* Peer-educators (*n=*42) chosen for “centrality,” rated highest by peers in communication ties and flow
*Interventions:* 5-A-Day message (to increase fruit and vegetable intake) plus accompanying 5-A-Day printed materials
*Findings:* Peer health educators were more likely to use “role modeling” (*p=*0.0004) and “creating context” (*p<*0.0001) as group change strategies, and “encouragement” (*p=*0.0009) and “responding to employee needs” (*p=*0.0001) were more likely to be used as individual change strategies
Strategies used differed with the sex of the educator; “mock competition,” “giving materials,” and “encouragement” were used by men significantly more than “creating context” and “keeping 5-A-Day visible”; women used “creating context” and “keeping 5-A-Day visible” significantly more than “mock competition”, “giving materials” and “encouragement” (*p<*0.0001 for all contrasts)
Hispanic peer health educators were more likely to use individual change strategies than their non-Hispanic counterparts (*p=*0.0128)
Patterson *et al.,* 1998 14
*Purpose:* Test whether the Working Well Trial nutrition intervention activities were maintained after the research program or were adopted by control sites
*Related papers:* Sorensen *et al.,* 1998 22
*Study design:* Randomized controlled trial
Dissemination of work-site smoking cessation interventions from the Working Well Trial to control cited at the end of the trial (*n=*54 control sites)
*Target group:* Worksite management
*Quality assessment:* Moderate
*Strategy evaluated:* Intervention materials were given to control sites (method not specified) at the conclusion of the Working Well Trial
*Interventions:* Nutrition interventions from the Working Well Trial
*Findings:* Only the findings pertaining to the control sites at the conclusion of the Working Well Trial are reported in this table
There was a significant increase in nutrition activity score (composite of nutrition classes or weight loss programs; self-help nutrition manuals and guides; videotapes, posters or brochures related to nutrition) from baseline to the end of the Working Well trial (2-year interval between start and completion of the trial; *p=*0.0012)
There was no significant increase on nutrition activity score in the control sites, between the end of the Working Well trial (point of dissemination of the nutrition interventions to control sites) and at the follow-up survey conducted 2 years
At follow-up, there was no significant difference between nutrition activity scores in the intervention compared with the control work sites
Samuels *et al.,* 1993 13
*Purpose:* Report on the first 3 years of the Project lean campaign, a national social marketing intervention, designed to promote dietary change
*Study design:* One group, post-intervention, process evaluation
*Target group:* General adult population
*Quality assessment:* Weak
*Strategy evaluated:* Media awareness campaign (television and print media)
*Interventions:* Telephone hotline which provided advice and offered an information booklet to callers
*Findings:* As a result of the ads and campaign publicity, the hotline received nearly 300,000 consumer calls
During the first 12 months of the campaign, calls peaked at 25,000–28,000 monthly; as publicity declined, so did calls to the hotline
Hotline was terminated after 18 months because of expense (more than US$300,000 annually)
Tziraki *et al.,* 2000 17
*Purpose:* Determine the effectiveness of two strategies for promoting the use of an nci nutrition manual by primary care physicians and their office staff
*Study design:* Randomized controlled trial
810 practices were randomized; 55 practices had a change in status and became ineligible after randomization
Workshop group (*n=*244): Practices received the manual and were invited to a training workshop
Postal delivery group (*n=*256): Practices were mailed the manual
Control group (*n=*255): Practices did not receive the manual
*Target group:* Free-standing primary care practices in Pennsylvania and New Jersey
*Quality assessment:* Weak
*Strategy evaluated:*
(1) Workshop: One staff member from each practice was invited to attend a 3-hour training session. Training was provided in the four major components of the manual—how to organize the office environment, how to screen patient adherence, how to provide dietary advice, and how to implement a patient follow-up system
(2) Postal delivery of the manual only (no training)
*Interventions:*nci sponsor nutrition manual for cancer prevention (contains multiple interventions)
Modelled after the nci publication *How to Help Your Patients Stop Smoking*
The manual addressed brief counselling techniques, office system organization, material resources, staff training, and patient educational materials
*Findings:* Follow-up interviews and observational assessments were conducted at 4–6 months post-dissemination with a physician and staff member from each practice
Adherence scores were calculated for four areas: office organization, nutrition screening, nutrition advice or referral, and patient follow-up
<50% of practices assigned to the workshop group sent representatives to the training workshop (120 of 244)
Workshop group was significantly more adherent to the manual’s recommendations for office organization at follow-up than the postal-delivery group (28.5% vs. 24.7%, *p<*0.005) and control group (28.5% vs. 23.0%, *p<*0.001); these analyses included all practices in the workshop group regardless of attendance at the training session.
Of those practices who attended the workshop, 30.6% were adherent to the recommendations for office organization
Workshop group was significantly more adherent to the manual’s recommendations for nutrition screening at follow-up than the postal-delivery group (23.5% vs. 21%, *p<*0.05) and control group (23.5% vs. 20.5%, *p<*0.05)
Of those practices attending the workshop, 25% were adherent to the recommendations for nutrition screening
No significant difference between the postal-delivery and control groups for office organization (24.7% vs. 23.0%) or nutrition screening (21% vs. 20.5%)
No statistically significant difference between the three groups for nutrition advice (workshop 54.9%; postal-delivery 53%; control 52.3%) nor for patient follow-up (workshop 14.6%; postal-delivery 13.6%; control 13.6%).
The attending workshop practices were significantly more likely than either postal-delivery (57% vs. 53%, *p<*0.05) or control groups (57% vs. 52.3%, *p<*0.05) to provide nutrition screening

a All located papers from U.S. sources.
